# Mama vs. Granny: An Exploratory fMRI Study on the Relative Roles of Female Caregivers in Children’s Cognitive and Emotional Outcomes

**DOI:** 10.3390/bs16071193

**Published:** 2026-07-15

**Authors:** Yunqing Ma, Wenrui Zhang, Bowen Hu, Xiujie Yang, Shaozheng Qin, Xiuyun Lin

**Affiliations:** 1 Institute of Developmental Psychology, Faculty of Psychology, Beijing Normal University, Beijing 100875, China; 2 State Key Laboratory of Cognitive Neuroscience and Learning & IDG/McGovern Institute for Brain Research, Beijing Normal University, Beijing 100875, China; 3 Beijing Key Laboratory of Applied Experimental Psychology, Faculty of Psychology, Beijing Normal University, Beijing 100875, China

**Keywords:** maternal caregiving, grandmaternal caregiving, working memory, emotional recognition, relative advantage

## Abstract

Previous research on the relative roles of maternal and grandmaternal caregiving in child development has been limited by the “maternal advantage hypothesis” and the “grandmaternal advantage hypothesis”. This study posited the “mother–grandmother relative advantage hypothesis” and aimed to evaluate the relative influence of maternal and grandmaternal caregiving in children’s cognitive and emotional outcomes at both behavioral and neural levels. This study comprised two components. In Study 1, a total of 33 children from mother-led or grandmother-led caregiving families were asked to perform a working memory task, aiming to investigate the behavioral and neural differences in cognitive outcomes between the two caregiving groups. In Study 2, a total of 31 children from similar family backgrounds were asked to complete an emotional recognition task, which was designed to examine the behavioral and neural differences in emotional outcomes between the groups. At a behavioral level, grandmaternal rather than maternal caregiving was associated with children’s working memory performance, whereas maternal rather than grandmaternal caregiving was associated with children’s emotional recognition performance. At a neural level, compared to children in the grandmaternal caregiving group, those in the maternal caregiving group showed increased functional connectivity with the right middle frontal gyrus and the left superior frontal gyrus as seeds during working memory tasks and increased functional connectivity with the right inferior occipital gyrus and left precentral gyrus as seeds during emotional recognition tasks. Our findings reveal a complementary pattern of female caregiving effects, supporting “mother–grandmother relative advantage hypothesis”.

## 1. Introduction

China’s labor market has undergone profound structural transformations, propelling dual-earner households to become the predominant family type (Cai et al., 2008). This societal shift has simultaneously reshaped child-rearing patterns and precipitated a contemporary parenting dilemma: with the increasing proportion of women in the workforce, childcare responsibilities have shifted to non-mother caregivers, particularly grandmothers (Chung et al., 2020; Luo et al., 2020). However, the misalignment between traditional experience-oriented grandmaternal caregiving and modern parenting demands may influence key developmental domains such as children’s cognitive and emotional competencies, thereby exacerbating maternal concerns and caregiving-related anxiety (Goh & Kuczynski, 2010). To address this growing societal challenge, a fundamental question should be answered: whether maternal and grandmaternal caregiving exert distinct effects on children’s cognitive and emotional outcomes. To answer this question, the present study recruited children from mother-led and grandmother-led caregiving families, requiring them to complete tasks assessing cognitive and emotional abilities while undergoing fMRI scans, aiming to provide behavioral- and neural-level insights into the aforementioned issue.

### 1.1. The Association Between Maternal and Grandmaternal Caregiving and Children’s Cognitive and Emotional Outcomes

Current research on the links between maternal/grandmaternal caregiving and children’s development presents two competing hypotheses. A body of research supports the “mother-vantage hypothesis”, which posits that when mothers serve as the primary caregivers, the influence of their caregiving generally fares better than that of grandmaternal caregiving. Hertwig et al. (2002) proposed three types of family resources that can support child development: material resources (e.g., financial means to ensure children’s access to food, healthcare, and education), cognitive resources (e.g., time investment in enhancing children’s academic performance), and interpersonal resources (e.g., emotional support). Influenced by parenting beliefs, mothers and grandmothers exhibit certain differences in the allocation of these three resource types. Specifically, mothers often assume a dual role as both “nurturers” and “educators”, simultaneously providing emotional support while maintaining high expectations for their children’s educational achievement and performance. This dual role motivates mothers to comprehensively invest in all three types of resources to the greatest extent possible. In contrast, grandparents typically exhibit intergenerational parenting characteristics such as “generational love” (gé bèi qīn), primarily focusing on daily caregiving—ensuring grandchildren’s basic living needs and physical well-being. Consequently, their resource allocation tends to be imbalanced, with excessive investment in material resources but insufficient allocation to cognitive and interpersonal resources. As a result, mothers instead of grandmothers may make a greater contribution to children’s cognitive and emotional functioning because they offer considerably greater cognitive and interpersonal resources. Indeed, the findings of Lu et al.’s (2022) study showed that children’s cognitive performance improved after parent–child but not grandparent–child interaction. Extending this line of evidence, a meta-analysis has further shown that grandparental caregiving is associated with poorer self-control among children (Zheng et al., 2025). Moreover, Qiu and Shum (2022) found that higher parental rather than grandparental expressive suppression led to lower emotion regulation competence in children.

Another body of studies supports the “grandmother-vantage hypothesis”, suggesting that grandmaternal caregiving may be more conducive to children’s cognitive development, emotional stability, and long-term survival adaptation. From an evolutionary perspective, the “grandmother effect” suggests that grandmothers improve the survival rate of their offspring by assisting in grandchild care (Hawkes et al., 1998). In other words, this evolutionary process has shaped a unique advantage in intergenerational parenting. Social reproduction theory posited by Bourdieu (1971) posits that compared with parents, grandparents have accumulated a certain level of cultural capital in society and established a wide social network, and they are able to flexibly utilize these resources to support their grandchildren, enabling the grandchildren to develop more effectively (Jaffee et al., 2011). Coupling with the low-pressure environment created by grandmothers, such a low-stress, high-responsive care may better align with children’s sensitive developmental periods and thereby promote more optimal growth in grandchildren. A study conducted in a sample of Chilean children revealed that compared to maternal care, children in grandparental care had higher cognitive, language, and motor scores (Narea et al., 2020). A recent study based on US families also indicated that grandparents could facilitate children’s cognitive development (Tompkins & Feng, 2025). The aforementioned indications shed light on a possibility: grandmothers might have advantages over mothers in improving the children’s cognitive and emotional development.

Both the “mother-vantage hypothesis” and the “grandmother-vantage hypothesis” suggest that maternal and grandmaternal caregiving differentially influence children’s cognitive and emotional outcomes. A critical question then arises: does this difference exhibit domain specificity/relative vantage? In terms of children’s cognitive outcomes, most studies have found the positive effects of grandmaternal caregiving on children’s cognitive abilities, yet few have explored its impact on working memory, a core cognitive component (Diamond, 2013). Working memory (WM) has a prolonged period of development (Ferguson et al., 2021) and is closely related to children’s academic achievement (Alloway & Alloway, 2010), mental and physical health (Lee et al., 2012; Riggs et al., 2010). Previous research has predominantly examined WM as a component of higher-order cognitive abilities, such as executive function, and investigated its relationship with maternal factors collectively (Bernier et al., 2012). Consequently, the unique influence of grandmaternal caregiving on WM and its distinction from maternal caregiving remain underexplored. Moreover, whether grandmothers contribute to children’s emotional development is still being debated. Chung et al. (2020) found that grandmother involvement was associated with improved 24-month-old children’s socioemotional behavior. Conversely, the results of Hansen and Hawkes’s (2009) study suggested that grandparental caregiving was related to a higher level of 36-month-old children’s emotional symptoms. Of the aspects of emotional function, emotion recognition (ER)—the ability to quickly process and accurately recognize emotions in others—is a key social skill that aids successful social interactions (Watling & Damaskinou, 2020). Given that children’s emotional function is profoundly influenced by primary caregivers (Dunsmore et al., 2009), the similarities and differences between maternal and grandmaternal caregiving in shaping children’s ER warrant further investigation to clarify caregiver-specific effects.

### 1.2. Neural Underpinnings of Maternal and Grandmaternal Caregiving Influences on Children’s Cognitive and Emotional Outcomes

Given the competitions in theories and empirical research, the mechanisms underlying the associations between maternal and grandmaternal caregiving and children’s cognitive and emotional outcomes warrant investigation. Investigating the neural substrates underlying the aforementioned associations enables us to clarify the inconsistent behavioral research findings on a more subtle level.

The brain regions related to WM and ER have been well documented. On the one hand, functional magnetic resonance imaging (fMRI) studies using cognitively demanding n-back working memory tasks have extensively documented the neural mechanisms underlying WM (Yaple & Arsalidou, 2018). The brain regions identified during the WM processing include the frontal regions, parietal cortex, and insula (Cao et al., 2021; Chang et al., 2013; H. Wang et al., 2019; Xu et al., 2023). On the other hand, considerable research has shown that face emotion recognition relies on a core set of brain regions, including the prefrontal cortex, anterior cingulate gyrus, amygdala, superior temporal gyrus, fusiform gyrus, and occipital cortex (Kennedy et al., 2021; Wiggins et al., 2016). Of those regions, the occipital cortex, particularly the inferior/middle occipital gyrus, is crucial for the initial stages of visual processing that contribute to the recognition of facial features and emotional expressions (Fairhall & Ishai, 2007). Previous studies suggested that abnormal activation in the aforementioned regions could lead to WM or ER deficits, and the interaction between different brain regions also marks critical significance for successful WM or ER processing (e.g., Cao et al., 2021; Nielsen et al., 2017; Yankouskaya et al., 2017).

Yet, the specific mechanism by which activation or interaction contributes to WM and ER under the context of maternal and grandmaternal caregiving remains unclear. Considerable maturation and reorganization in brain happen in late childhood and continue to late adolescence (Sowell et al., 2002). Such changes allow the brain to remain plastic and are thought to give chances for environmental input to shape the development of the brain system and to impact related cognition or emotion (Drzewiecki & Juraska, 2020). Of the multiple environmental factors, caregivers may represent the core and proximal factor of the child’s environment, which has the ability to enhance or impair the development of children’s cognitive and emotional outcomes.

Most researchers focus on the child-driven effect, that is, the activation of maternal and non-maternal brains by stimuli from children. For example, Bjertrup et al. (2021) found that when mothers view pictures of their own as opposed to unknown children, they displayed increased neural activation in the insula, dorsolateral prefrontal cortex, and occipital brain areas. At the same time, Rilling et al. (2021) first observed such an effect among non-mothers, including grandmothers. They found that when viewing pictures of their grandchildren, grandmothers showed strong neural activation in the temporo–parietal junction and dorsomedial prefrontal cortex. These inconsistent results reflect differences in how mothers and grandmothers process stimuli from children, which subsequently may manifest through the way they treat children. Thus, the caregiver-driven effect (i.e., activation of children’s brain by stimuli from caregivers) should also be considered. To date, a few studies have demonstrated that maternal caregiving affected children’s neural response related to cognitive and emotional processing. Specifically, Romund et al. (2016) found that when children completed a negative emotional face recognition task, an increased amygdala response was correlated with maternal parenting. Moreover, the findings of a study conducted by Dandash et al. (2021) found that maternal behavior affected children’s brain activation related to cognitive processing, such as the left parietal and dorsolateral prefrontal cortex. Although grandmothers are increasingly recognized as caregivers and attachment figures to their grandchildren, neuroimage studies focusing on or including the impacts of grandmothers on children are scarce. Nonetheless, inconsistencies in aforementioned behavioral findings hint that grandmother–grandchild relationships may be characterized by similar but not necessarily equal neural activation as compared to mother–child relationships.

Moreover, Pozzi and his colleagues conducted a series of studies and revealed that maternal caregiving also impacted functional connectivity (FC) between brain regions and even networks among children (Pozzi et al., 2021; Pozzi et al., 2024). Recently, especially, they pointed out that maternal parenting was associated with changed FCs between brain regions of several large-scale networks implicated in children’s cognitive and affective processing, including the ventral attention network, the frontoparietal network, and the default mode network. Noting that Pozzi’s study primarily focused on resting-state FCs, another question arises: are there similar FCs under the circumstances of specific cognitive or affective tasks? And do maternal and grandmaternal caregiving affect these FCs differently? Given the above, the second aim of the present study is to compare the differences in brain activation and FCs between children influenced by maternal and grandmaternal caregiving while completing emotional and cognitive processing tasks. Considering that our study is exploratory, psychophysiological interaction (PPI) analysis, a data-driven method, allows for full analysis of the context-dependent functional interaction among brain regions in specific cognitive or emotional tasks for children in the maternal or grandmaternal caregiving groups.

### 1.3. The Present Study

The primary objective of this study was to examine, at both the behavioral and neural levels, the relative associations of maternal versus grandmaternal caregiving with children’s cognitive and emotional development. It should be noted that research comparing these two caregiving configurations in relation to children’s neurodevelopment remains extremely scarce, providing insufficient empirical grounding for formulating directional hypotheses a priori. Accordingly, all analyses in the present study were positioned as exploratory in nature. Under this premise, we did not prespecify directions; instead, we aimed to generate preliminary, testable hypotheses that may inform future investigations in this nascent field.

## 2. Study 1: Behavioral and Neural Differences in Children’s Cognitive Outcome

### 2.1. Participants and Procedures

We recruited children from mother-led caregiving families or grandmother-led caregiving families to participate in an fMRI assessment. With reference to the study of Tang et al. (2024), the specific screening criteria for mother-led caregiving families include that (1) they had a child between the ages of 8 and 10, (2) the child always live with their mothers, and (3) grandmothers occasionally help with babysitting, but do not take responsibility for the care and upbringing of children. Families were designated as grandmother-led caregiving families when meeting the following criteria: (1) had a child between the ages of 8 and 10, (2) the child had lived with their grandmother for more than half of the child’s life since birth, and (3) the target child had lived with their grandmother for more than half the time in the past year. Families in which parents were divorced, reported substance misuse, or were widowed were excluded from this study.

Using G*Power 3.1 (ANOVA: Repeated Measures, Within-Between Interaction), a total sample size of 28 participants was determined for a two-factor mixed experimental design, with an effect size of 0.25, a significance level of 0.05, and a statistical power of 0.80. Study 1 met this criterion. A total of 33 children completed the digit n-back task (48.5% girls; maternal caregiving group *n* = 18, *M*_age_ = 8.96 years old, *SD* = 0.81; grandmaternal caregiving group *n* = 15, *M*_age_ = 9.63 years old, *SD* = 1.45). All children met the inclusionary criteria for children determined through the child and parent report, including diagnosis of a neurological developmental disorder, head injury, use of pharmaceuticals that impact central nervous system functioning, and contraindications to MRI. Each family pair (mother–child dyads and grandmother–grandchild dyads) signed an informed consent form prior to the fMRI scanning. The research procedures were approved by the Institutional Review Board of Department of Psychology at the corresponding university. Upon completion of all study tasks, each family pair will be paid for their participation.

### 2.2. Digit n-Back Task

Qin et al. (2009) provided a classic paradigm for studying working memory. Adapted from it, the digit n-back task was applied, which used a blocked-design including 12 cycles of alternating blocks: four were 0-back blocks, four were 1-back blocks and four were 2-back blocks. The 0-, 1-, or 2-back condition was signaled by a 2 s cue presentation at the beginning of each block, which comprised 15 randomly chosen single digits shown serially for 0.4 s each, separated by a 1.4 s interstimulus interval. Children were asked to detect whether the current item was “1” (0-back condition), or had appeared one (1-back condition) or two (2-back condition) positions back in sequence.

### 2.3. Image Data Acquisition

Structural and functional MRI data were collected with a 3T Siemens Trio Tim scanner (Erlangen, Germany) using a whole brain echo-planar imaging sequence. Thirty-three axial slices (4 mm thickness, 0.6 mm skip) were imaged with the following parameters: repetition time (TR) = 2000 ms, echo time (TE) = 30 ms, flip angle (FA) = 90°, voxel size = 3.125 × 3.125 × 4 mm^3^, field of view (FOV) = 200 × 200 mm^2^.

High-resolution anatomical images of each participant were acquired using three-dimensional sagittal T1-weighted magnetization-prepared rapid gradient echo with 192 slices: TR = 2530 ms, TE = 3.45 ms, FA = 7°, inversion time (TI) = 1100 ms, voxel size = 1 × 1 × 1.0 mm^3^, matrix size = 256 × 256, FOV = 256 × 256 mm^2^, bandwidth (BW) = 190 Hz/Px, slice thickness = 1 mm.

Stimulus were shown and responses were recorded using E-prime 2.0 (Psychology Software Tools, Inc., Pittsburgh, PA, USA).

### 2.4. Behavioral Data Analysis

Repeated-measures ANOVAs were conducted for both the digit n-back task, with condition (0-back vs. 1-back vs. 2-back) as within-subject factor and group (maternal caregiving vs. grandmaternal caregiving) as between-subject factor. SPSS 22.0 was used for the above analyses.

### 2.5. Imaging Data Analysis

#### 2.5.1. Imaging Preprocessing

The Statistical Parametric Map 12 (SPM12) software based on MATLAB 20.0 platform was used for image preprocessing. For signal stabilization and participants’ adaptation to scanning noise, the first 4 functional volumes were discarded. Then, remaining images underwent the following common preprocessing steps: first, images were corrected for slice acquisition timing and realigned for head motion correction; next, they were coregistered to each participants’ gray matter image segmented from corresponding high-resolution T1-weighted image; then, the realigned images were spatially normalized into a standard stereotactic space of the Montreal Neurological Institute (MNI) space and resampled into 2 mm isotropic voxels; finally, images were spatially smoothed by convolving an isotropic 3D-Gaussian kernel with 6 mm full width at half maximum (FWHM).

#### 2.5.2. Activation Analyses

To identify areas engaged by the task, an activation analysis was performed. This was used to identify volumes of interest (VOIs) used as seeds in the PPI analyses.

Task conditions (0-back, 1-back, and 2-back) were modeled separately as regressors based on a general linear model. Additionally, realignment parameters were included to account for movement-related variability. To identify areas activated by the task, individual contrast images (1-back > 0-back, 2-back > 0-back) were computed, respectively. One-sample *t*-tests were applied to analyze the differences within the group, and two-sample *t*-tests were used to compare differences between the two groups in SPM 12. FWE correction *p* < 0.05 was used.

#### 2.5.3. Psychophysiological Interaction Analyses

The PPI analyses were performed to measure the correlations of time series of VOIs with other brain regions. Because the activation analysis did not show any between-group differences, we selected VOIs with reference to Nielsen et al. (2017), based on an effect across groups for the 2-back > 0-back contrast, including left medial superior frontal cortex (MSFG: x = −4, y = 24, z = 44), the bilateral anterior insula (left AI: x = −30, y = 24, z = −2; right AI: x = 32, y = 26, z = −2), the left superior frontal gyrus (SFG; x = −26, y = 8, z = 62), the left inferior parietal lobule (IPL; x = −28, y = −50, z = 42), and the right middle frontal gyrus (MFG; x = 28, y = 10, z = 54).

Aforementioned brain areas were chosen as VOIs and time series were extracted. This was accomplished by extracting the first eigenvariate from a sphere with a radius of 6 mm, starting from the group-level peak coordinates and moving the center of the sphere to the nearest local maximum within 10 mm masking at *p* < 0.05 (uncorrected) (Nielsen et al., 2017). The design matrix of PPI consisted of three main regressors: the physiological variable that represents the time series from VOIs, the psychological variable which represents the task conditions (WM, created using a 2-back > 0-back contrast), and the PPI variable. Finally, the results of PPI of all participants were extracted for group-level analysis. FC between each seed and other brain areas was analyzed separately. A cluster-level FWE correction *p* < 0.05 with a voxel-level threshold of *p* < 0.001 was used.

#### 2.5.4. Correlation Analyses

For each participant, we extracted the beta value of FC between brain regions, which showed between-group differences at the condition of WM using RESTplus v1.30 (http://restfmri.net/forum/ (accessed on 15 July 2024)). Then, the partial correlation analyses were performed to evaluate the relationship to the accuracy scores and reaction times (RT) of the working memory task.

### 2.6. Results

#### 2.6.1. Behavioral Results

Two separate repeated-measures ANOVAs for accuracy and reaction times (RTs) were conducted with WM loads (0-back vs. 1-back vs. 2-back) as within-subject factor and group (maternal caregiving vs. grandmaternal caregiving) as between-subject factor. After controlling for children’s gender and age, there were robust main effects of WM loads on both accuracy (*F*_(2,58)_ = 4.38, *p* = 0.017, η^2^_p_ = 0.131) and RTs (*F*_(2,58)_ = 7.92, *p* = 0.002, η^2^_p_ = 0.215). Post hoc analysis indicated that increasing WM loads were associated with reduced accuracy and increased RTs. We only found a significant group × WM-load interaction for accuracy (*F*_(2,58)_ = 6.13, *p* = 0.004, η^2^_p_ = 0.174) but not for RTs (*F*_(2,58)_ = 0.65, *p* = 0.526, η^2^_p_ = 0.022). Simple effect analysis indicated that children in the grandmaternal caregiving group (*M* = 0.74, *SD* = 0.05) displayed higher accuracy in 2-back condition than children in the maternal caregiving group (*M* = 0.56, *SD* = 0.05), while there were no significant differences in 0- and 1-back condition between the maternal (0-back: *M* = 0.96, *SD* = 0.02; 1-back: *M* = 0.95, *SD* = 0.02) and grandmaternal (0-back: *M* = 0.96, *SD* = 0.02; 1-back: *M* = 0.92, *SD* = 0.02) caregiving group. We did not reveal any significant between-group differences for either accuracy (*F*_(1,29)_ = 2.21, *p* = 0.148, η^2^_p_ = 0.071) or RTs (*F*_(1,29)_ = 0.29, *p* = 0.598, η^2^_p_ = 0.010).

#### 2.6.2. Brain Activation

Task analysis showed significant activation of WM in the left MSFG, bilateral AI, left SFG, left IPL, and right MFG (Table 1). However, no significant group differences in activation were observed (*p*FWE < 0.05).

#### 2.6.3. Functional Connectivity Results

**Figure 1:**
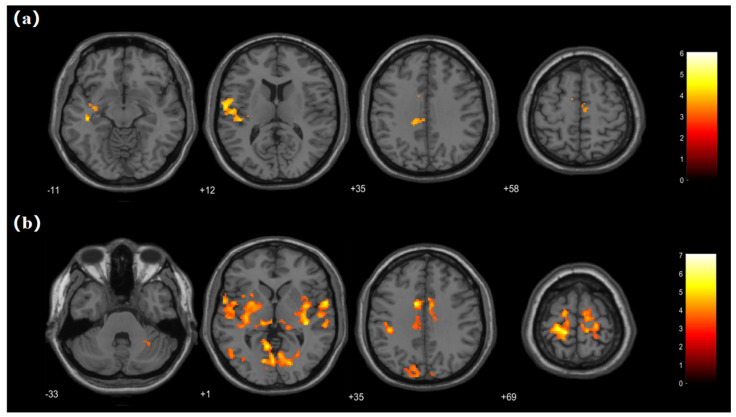
Between-group differences (maternal caregiving > grandmaternal caregiving) in PPI analysis during digit n-back task at *p* < 0.05 (FWE corrected at cluster level) with a voxel-level threshold of *p* < 0.001 (uncorrected). (**a**) Significant group differences in PPIs using the right MFG as a seed. (**b**) Significant group differences in PPIs using the left SFG as a seed. MFG, middle frontal gyrus; SFG, superior frontal gyrus.

With the right MFG as a seed region, children in the grandmaternal caregiving group showed significantly decreased FCs between the right MFG and left middle cingulate gyrus and left heschl gyrus compared with children in the maternal caregiving group. Meanwhile, with the left SFG as a seed region, children in the grandmaternal caregiving group showed significantly decreased FCs between the left SFG and the left postcentral gyrus, left lingual gyrus, bilateral middle temporal gyrus, and the left superior occipital gyrus compared with children in the maternal caregiving group (Figure 1, Table 2). No significant group differences in FC were found with other VOIs as seeds.

#### 2.6.4. Correlation with Behavioral Performance

The present study only found a significant positive correlation in FC between the right MFG and the right middle temporal gyrus and the accuracy score of 2-back condition (*r* = 0.60, *p* = 0.030) in the grandmaternal caregiving group but not in the maternal caregiving group.

## 3. Study 2: Behavioral and Neural Differences in Children’s Emotional Outcome

### 3.1. Participants and Procedures

The inclusion criteria for maternal and grandmaternal caregiving families are the same as in Study 1. Using G*Power 3.1 (ANOVA: Repeated Measures, Within-Between Interaction), a total sample size of 34 participants was determined for a two-factor mixed experimental design, with an effect size of 0.25, a significance level of 0.05, and a statistical power of 0.80. Study 2 was marginally below this estimated target. A total of 31 children (48.4% girls; maternal caregiving group *n* = 15, *M*_age_ = 9.87 years old, *SD* = 1.51; grandmaternal caregiving group *n* = 16, *M*_age_ = 8.86 years old, *SD* = 0.95) completed the negative emotional face matching task. All of children met the inclusionary criteria, which were the same as those Study 1. Each family pair (mother–child dyads and grandmother–grandchild dyads) signed an informed consent form prior to the fMRI scanning. The research procedures were approved by the Institutional Review Board at the corresponding university. Upon completion of all study tasks, each family pair will be paid for their participation.

### 3.2. Negative Emotional Face Matching Task

The negative emotional face matching task was adapted from Hariri et al. (2002). This task consisted of 10 blocks: five were emotional face blocks and five were scrambled shape blocks. The emotional face or scrambled shape was signaled by a 5 s cue presentation at the beginning of each block, and then followed by 6 trials of images with 5 s each. During the emotional face condition, children selected one of two negative facial expressions in the bottom that expressed the same emotion as the target expression on the top. Negative facial expressions (i.e., fear, anger) were included and obtained from a standardized dataset of Chinese faces (Y. Wang & Luo, 2005). During the scrambled shape condition, children selected one of two scrambled shapes in the bottom that displayed the same orientation as the target scrambled shape.

### 3.3. Image Data Acquisition

Following the same procedure as Study 1.

### 3.4. Behavioral Data Analysis

Repeated-measures ANOVAs were conducted for both the negative emotional face matching task, with condition (emotional face vs. scrambled shapes) as within-subject factor and group (maternal caregiving vs. grandmaternal caregiving) as between-subject factor. SPSS 22.0 was used for above analyses.

### 3.5. Imaging Data Analysis

#### 3.5.1. Imaging Preprocessing

Following the same procedure as Study 1.

#### 3.5.2. Activation Analyses

To identify areas engaged by the task, an activation analysis was performed. This was used to identify VOIs used as seeds in the PPI analyses. Task conditions (emotional face and scrambled face) were modeled separately as regressors based on a general linear model. Additionally, realignment parameters were included to account for movement-related variability. To identify areas activated by the task, individual contrast images (emotional face > scrambled face) were computed, respectively. One-sample *t*-tests were applied to analyze the differences within the group, and two-sample *t*-tests were used to compare differences between the two groups in SPM 12. FWE correction *p* < 0.05 was used.

#### 3.5.3. Psychophysiological Interaction Analyses

The PPI analyses were performed to measure the correlations of time series of VOIs with other brain regions. The present study found that significant ER (emotional face > scrambled face) across all participants existed in the left inferior occipital gyrus (left IOG; x = 32, y = −92, z = −10), right middle occipital gyrus (right MOG: x = −24, y = −94, z = 0), the right opercular part of the inferior frontal gyrus (OpIFG; x = 42, y = 14, z = 28), and left precentral gyrus (PreCG; x = −42, y = 6, z = 30). Aforementioned brain areas were chosen as VOIs and time series were extracted. This was accomplished by extracting the first eigenvariate from a sphere with a radius of 6 mm, starting from the group-level peak coordinates and moving the center of the sphere to the nearest local maximum within 10 mm masking at *p* < 0.05 (uncorrected). Moreover, the design matrix of PPI consisted of three main regressors: the physiological variable that represents the time series from VOIs, the psychological variable which represents the task conditions (ER, created using an emotional face > scrambled face contrast), and the PPI variable. Finally, the results of PPI of all participants were extracted for group-level analysis. FC between each seed and other brain areas was analyzed separately. A cluster-level FWE correction *p* < 0.05 with a voxel-level threshold of *p* < 0.001 was used.

#### 3.5.4. Correlation Analyses

For each participant, we extracted the beta value of FC between brain regions, which showed between-group differences at the condition of ER using RESTplus v1.30 (http://restfmri.net/forum/ (accessed on 15 July 2024)). Then, the partial correlation analyses were performed to evaluate the relationship to the accuracy scores and reaction times (RT) of the emotion recognition task.

### 3.6. Results

#### 3.6.1. Behavioral Results

Two separate repeated-measures ANOVAs for accuracy and reaction times (RTs) were conducted with shape (emotional face vs. scrambled shape) as within-subject factor and group (maternal caregiving vs. grandmaternal caregiving) as between-subject factor. After controlling for children’s gender and age, there was no robust main effect of shape on accuracy (*F*_(1,27)_ = 3.19, *p* = 0.085, η^2^_p_ = 0.106). There was a significant group × shape interaction for accuracy (*F*_(1,27)_ = 5.42, *p* = 0.028, η^2^_p_ = 0.167). Simple effect analysis indicated that children in the grandmaternal caregiving group (*M* = 0.61, *SD* = 0.03) displayed lower accuracy in emotional face condition than children in the maternal caregiving group (*M* = 0.69, *SD* = 0.03), while there was no significant difference in scrambled face condition between maternal (*M* = 0.74, *SD* = 0.02) and grandmaternal (*M* = 0.76, *SD* = 0.02) caregiving groups. For RTs, there was a significant main effect of shape (*F*_(1,27)_ = 13.97, *p* = 0.001, η^2^_p_ = 0.341). Post hoc analysis indicated that compared to scrambled face condition, emotional face condition was associated with increased RTs. There was no significant group × shape interaction for RTs (*F*_(1,27)_ = 0.31, *p* = 0.580, η^2^_p_ = 0.011). We did not reveal any significant between-group differences for either accuracy (*F*_(1,27)_ = 1.65, *p* = 0.210, η^2^_p_ = 0.058) or RTs (*F*_(1,27)_ = 0.16, *p* = 0.695, η^2^_p_ = 0.006).

#### 3.6.2. Brain Activation

Task analysis showed significant activation in the left IOG, right MOG, right OpIFG, and left PreCG (Table 3). No significant group differences were observed (*p*FWE < 0.05).

#### 3.6.3. Functional Connectivity Results

**Figure 2:**
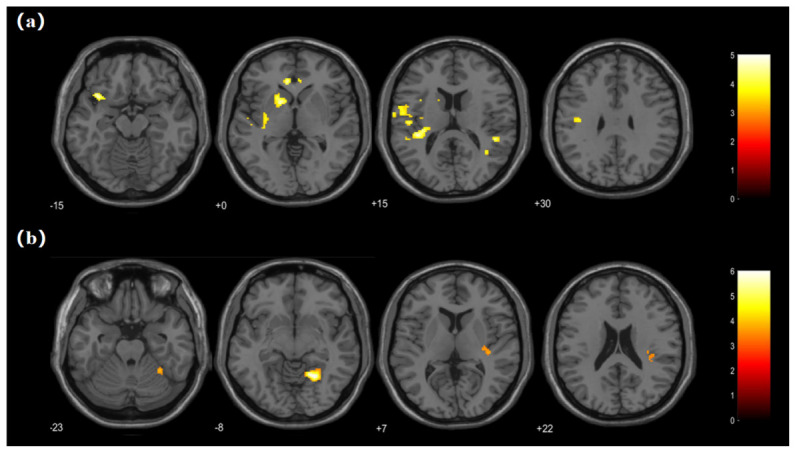
Between-group differences (maternal caregiving > grandmaternal caregiving) in PPI analysis during digit negative emotional face matching task at *p* < 0.05 (FWE-corrected at cluster level) with a voxel-level threshold of *p* < 0.001 (uncorrected). (**a**) Significant group differences in PPIs using the right IOG as a seed. (**b**) Significant group differences in PPIs using the left PreCG as a seed. IOG, inferior occipital gyrus; PreCG, precentral gyrus.

With the right IOG as a seed region, children in the grandmaternal caregiving group showed significantly decreased FCs between the right IOG and the left rolandic operculum, and the left orbital part of the inferior frontal gyrus, and bilateral superior temporal gyrus compared with children in the maternal caregiving group. With the left PreCG as a seed region, children in the grandmaternal parenting group showed significantly decreased FCs between the left PreCG and the right lingual gyrus, and right putamen compared with children in the maternal caregiving group (Figure 2, Table 4). No significant group differences in FC were found with other VOIs as seeds.

#### 3.6.4. Correlation with Behavioral Performance

The present study only found a significant positive correlation in FC between the right IOG and the left superior temporal gyrus and the accuracy of shape condition (*r* = 0.56, *p* = 0.047) in the maternal caregiving group but not in the grandmaternal caregiving group.

## 4. Discussion

This study is novel in that it investigates the relative roles of maternal and grandmaternal caregiving in children’s cognitive (i.e., working memory) and emotional (i.e., emotion recognition) outcomes at both behavioral and neural levels. Regarding WM, grandmaternal rather than maternal caregiving was significantly associated with children’s WM performance; decreased FCs with the right MFG and the left SFG as seeds during WM tasks were found in the grandmaternal caregiving group. Regarding ER, maternal rather than grandmaternal caregiving was significantly associated with children’s ER performance; decreased FCs with the right IOG and left PreCG as seeds during ER tasks were found in the grandmaternal caregiving group. In other words, grandmaternal caregiving demonstrated a specific/relative advantage in enhancing children’s cognitive abilities, whereas maternal caregiving showed a relative advantage in supporting emotional competences. These findings led us to propose the “mother-grandmother relative vantage hypothesis”, highlighting the need to incorporate the distinct functional roles of mothers and grandmothers in child development when designing family caregiving support systems.

### 4.1. The Relative Roles of Maternal and Grandmaternal Caregiving in Children’s Cognitive Outcome

Consistent with the findings of Narea et al. (2020), we found that children in the grandmaternal caregiving group outperformed those in the maternal caregiving group in the digital n-back task, demonstrating a relative advantage in high-cognitive-load conditions. From the perspective of intergenerational cultural capital transmission (Bourdieu, 1971), grandmothers, as key intergenerational caregivers, often possess more accumulated cultural capital than mothers. This is particularly evident in our sample—drawn from urban Chinese families where three-generation co-residence remains a culturally normative childcare arrangement—grandparents’ socioeconomic and educational advantages are particularly pronounced (National Bureau of Statistics, 2021). Consequently, they can provide grandchildren with enriched cognitive stimulation (e.g., reading, play-based activities, and verbal interactions) and higher-quality educational resources (e.g., extracurricular tutoring, cultural exposure). This intergenerational transfer of cultural capital likely contributed to the enhanced performance of children from grandmother-led families in the n-back task. Regarding caregiving styles, grandmothers tend to exhibit higher emotional responsiveness and lower control compared to mothers, who may adopt stricter disciplinary approaches (Coall & Hertwig, 2010). Such a low-pressure, high-support caregiving environment may reduce children’s cognitive load stress, enabling more efficient allocation of working memory resources during demanding tasks (e.g., the 2-back condition). Furthermore, grandmothers’ ample caregiving experience may equip them with adaptive strategies to better support children’s cognitive challenges, thereby optimizing task performance. Nevertheless, these positive features coexist with well-documented challenges in grandparental caregiving, including intergenerational conflicts, divergent educational philosophies, and physical-emotional strain (Coall & Hertwig, 2010; Xiao, 2016), all of which are salient in Chinese three-generation households and may attenuate the very benefits described above. The present findings should therefore be situated within the specific context of our urban, socioeconomically advantaged sample, where such stressors may be relatively minimized.

The neuroimaging findings of the present study further revealed that, compared to the maternal caregiving group, children in the grandmaternal caregiving group exhibited significantly weaker FCs with seed points in the right MFG and the left SFG. The involved brain regions include the cingulate gyrus (a key hub for cognitive control and emotion regulation; Liu et al., 2021), middle temporal gyrus (a core area for semantic memory and multimodal information integration; Onitsuka et al., 2004), and occipital lobe (a higher-order cortex for visual information processing; Kozlovskiy et al., 2014). Previous studies have confirmed that FCs in these regions, particularly changes in frontal-cingulate connectivity, were closely associated with WM performance (Huang et al., 2024). Notably, when considered alongside behavioral results, this weakened FCs does not reflect cognitive processing deficits but may instead indicate a more efficient neural network operation pattern. This pattern is particularly evident in the functional connectivity between the right MFG and the right middle temporal gyrus, which shows a significant positive correlation with working memory behavioral performance. Critically, this positive correlation, combined with the overall reduced connectivity in this group and comparable behavioral outcomes, supports the interpretation that the neural system operates in a cost-efficient manner rather than simply reflecting functional impairment. Several mechanisms could potentially underlie this optimized neural pattern in the context of grandmaternal caregiving: (1) intergenerational transmission of cultural capital promotes the development of more mature cognitive strategies; (2) low-pressure parenting approaches decrease competitive resource allocation by the limbic system; and (3) abundant early language stimulation may enhance the synergistic efficiency between the default mode network and working memory network. These findings provide preliminary empirical evidence for understanding how intergenerational parenting differences may influence children’s neural development. Specifically, during high cognitive-load tasks, the enhanced functional connectivity observed in the maternal caregiving group may indicate greater neural resource mobilization, whereas the reduced connectivity in the grandmaternal caregiving group is more consistent with a “low-cost, high-efficiency” neural pattern. This interpretation requires longitudinal and multimodal validation; it also warrants acknowledgment that the apparent neural efficiency may be moderated by caregiving-related stressors, such as intergenerational discord and caregiver burden, which were not systematically assessed here and await further investigation across diverse family contexts.

### 4.2. The Relative Roles of Maternal and Grandmaternal Caregiving in Children’s Emotional Outcome

At the behavioral level, children in the maternal caregiving group outperformed their peers in the grandmaternal caregiving group in the negative emotional face matching task, indicating a relative advantage in ER under negatively valenced conditions. This finding aligns with prior longitudinal evidence suggesting that maternal care is more consistently associated with children’s accurate interpretation of emotional cues, particularly threat-related expressions (Lu et al., 2022; Qiu & Shum, 2022). One possible explanation is that maternal caregivers tend to provide more emotionally contingent and elaborative responses to children’s distress, thereby facilitating the differentiation and labeling of negative emotions (Sadri & Yates, 2025). In contrast, grandmaternal caregiving, while often emotionally supportive, may offer fewer structured opportunities for children to practice identifying and responding to negative affective signals, potentially due to generational differences in emotional socialization practices (Barnett et al., 2010). This difference likely reflects generational divergence in emotion socialization practices (Barnett et al., 2010) as enacted within contemporary urban Chinese families. In this cultural context, intensive mothering norms prescribe high-contingency emotional coaching from early childhood, whereas grandmothers’ roles have been historically shaped by collectivist family values that emphasize practical caregiving contributions and family cohesion over explicit emotion-teaching.

Beyond behavioral differences, neuroimaging analyses revealed more nuanced group disparities in FC involving two seed regions: significant group disparities emerged in FCs with seed regions in the right IOG (a core area for facial emotion processing; Fairhall & Ishai, 2007) and left PreCG (critical for facial perception and emotion recognition; Leshin et al., 2024; Stuhrmann et al., 2011). Specifically, children in the maternal care group showed stronger positive coupling between the right inferior occipital gyrus and bilateral superior temporal gyri, regions implicated in recognizing and integrating emotional information during social communication (Belin et al., 2004), as well as with the inferior frontal gyrus, a key node in emotion regulation processes (Ewell et al., 2023). Additionally, the maternal care group exhibited enhanced connectivity from the left precentral gyrus to the right lingual gyrus, involved in visual emotion processing, and to the basal ganglia, which contributes to emotional valence evaluation (Leshin et al., 2024; Stuhrmann et al., 2011).

These neural patterns, converging with the behavioral advantage favoring maternal caregiving, suggest that early caregiving context may shape not only the accuracy of emotion recognition but also the efficiency of distributed neural networks supporting affective perception and regulation. This pattern is particularly evident in the FC between the right IOG and the left superior temporal gyrus, which shows a significant positive correlation with emotion recognition performance. One interpretation is that maternal care, characterized by greater responsiveness and emotional availability, may promote the integration of visual emotion processing regions, such as the inferior occipital gyrus and lingual gyrus, with higher order social cognitive and regulatory circuits, including the superior temporal gyri and inferior frontal gyrus. Such integration could enhance children’s ability to detect and interpret negative emotions while simultaneously recruiting regulatory mechanisms to maintain goal-directed behavior (Ochsner & Gross, 2005). Conversely, the relatively weaker connectivity of children from grandmother-led families in these circuits may reflect less frequent or less contingent emotional exchanges, which could impede the automatic recruitment of these networks during emotionally demanding tasks. These findings extend prior work by clarifying that the impact of alternative caregiving arrangements on children’s emotional development is not merely behavioral but is also instantiated at the level of large-scale brain network organization.

### 4.3. The Proposed “Relative Vantage Hypothesis” and Its Implication

**Figure 3:**
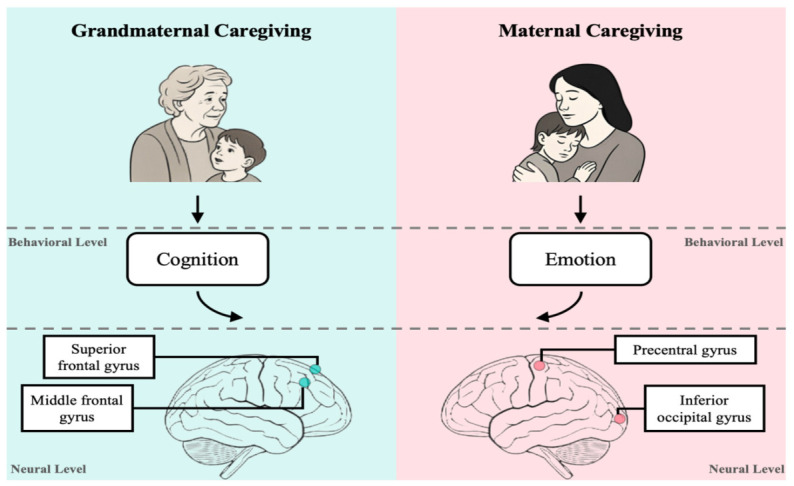
Theoretical framework of the mother–grandmother relative advantage hypothesis. At the behavioral level, grandmaternal caregiving was associated with enhanced cognitive performance, whereas maternal caregiving was associated with emotional outcomes. At the neural level, distinct brain regions showed differential involvement between groups.

The findings regarding children’s cognitive and emotional outcomes do not overwhelmingly favor one parenting style over the other. Instead, they reveal a pattern of relative vantages. Accordingly, the current findings tentatively suggest a “mother–grandmother relative advantage” framework, which posits that maternal and grandmaternal caregiving exhibit distinct relative strengths across different developmental domains (Figure 3). Specifically, grandmothers demonstrate greater advantages in promoting children’s cognitive development compared to mothers, whereas mothers show relatively stronger benefits for children’s emotional competence. This preliminary framework, which moves beyond the traditional binary view of caregiving superiority by highlighting the complexity and complementarity of intergenerational parenting effects, offers a heuristic lens for generating future formal theoretical claims. Future research should address two key questions: (1) the developmental timing of parenting advantages and their associated biomarkers, with longitudinal designs poised to examine whether neural and hormonal correlates systematically covary with the domain-specific advantage pattern; and (2) the design of cross-generational intervention programs that leverage these complementary strengths, providing an avenue to test whether harnessing grandmothers’ cognitive contributions and mothers’ emotional support yields synergistic benefits beyond either approach alone. Collectively, this framework provides an initial perspective for understanding child development in multigenerational families, pending replication and extension in future work.

Practically, these exploratory findings hint that grandmothers’ unique and positive contribution to children’s cognitive development may ease some parenting anxieties among mothers. These findings tentatively call for a reevaluation of traditional family support systems and suggest the need to establish more comprehensive tri-generational collaborative caregiving networks. Specifically, parenting interventions and early education policies might consider actively encouraging grandmothers’ participation in fostering children’s cognitive development, while emphasizing mothers’ supportive roles in emotional socialization. From both behavioral and neural perspectives, this study provides initial insights into the differential effects of maternal and grandmaternal caregiving on children’s cognitive and emotional abilities. Importantly, it preliminarily highlights the crucial role of grandparents in child development, offering a more nuanced understanding of intergenerational parenting dynamics.

### 4.4. Limitations and Future Directions

This study has several limitations that should be acknowledged. First, the relatively small sample size may affect the robustness of the findings, and these findings should be viewed as exploratory and hypothesis-generating rather than conclusive, and await replication in larger samples. Second, the study could not examine potential differences between paternal and maternal grandmothers’ caregiving roles. Given that factors such as genetic relatedness, co-residence patterns, and emotional bonds may lead to variations in caregiving involvement, parenting styles, and grandchild–grandparent closeness between paternal and maternal grandmothers, future studies should explore these distinctions more thoroughly. Third, while the study controlled for children’s demographic variables, it did not account for caregiver-related factors such as family socioeconomic status, maternal and grandmaternal age, health status, or educational attainment, which may influence parenting practices and, consequently, children’s cognitive and emotional outcomes. Future research should incorporate these variables to better isolate the specific effects of maternal and grandmaternal caregiving on child development. Fourth, given the cross-sectional design, all associations are correlational rather than causal. Additionally, we lacked complementary behavioral or psychophysiological measures (e.g., parenting assessments, attachment evaluations, EEG/ERP, eye-tracking) that could provide convergent evidence for the mechanisms underlying our fMRI findings. These combined limitations highlight the need for future longitudinal studies incorporating multi-method assessments to better capture the dynamic interplay between grandparental caregiving and child development, as well as the underlying neural mechanisms. Fifth, the present study grouped children based on their primary caregiver for analytical purposes, which does not fully capture the fact that caregiving in Chinese families often involves multiple figures. The observed differences likely reflect broader family caregiving constellations rather than the exclusive effect of a single caregiver. Future studies with continuous measures of caregiving involvement across family members would help clarify these dynamics.

## 5. Conclusions

This exploratory study provides preliminary evidence suggesting potential domain-specific patterns associated with maternal versus grandmaternal caregiving. Specifically, grandmaternal caregiving showed associations with better working memory performance and reduced frontal connectivity, which may tentatively suggest more efficient neural processing under high cognitive load, whereas maternal caregiving showed associations with better emotion recognition and stronger connectivity within emotion-related circuits. Rather than claiming superiority of one caregiving type over another, we suggest that different caregiving configurations may be associated with distinct cognitive and neural profiles, a hypothesis that awaits rigorous testing in future research and carries important implications for early education policy and family support systems in an era of increasing multigenerational caregiving arrangements.

## Figures and Tables

**Table 1:** Brain activation during digit n-back task (2-back > 0-back) across all participants.

Brain Region	MNI Coordinate			*k*	*t*
	*x*	*y*	*z*		
L. Medial superior frontal cortex	−4	24	44	582	10.04
L. Anterior insula	−30	24	−2	256	9.90
R. Anterior insula	32	26	−2	234	9.51
L. Superior frontal gyrus	−26	8	62	312	8.47
L. Inferior parietal lobule	−28	−50	42	309	8.28
R. Middle frontal gyrus	28	10	54	352	8.26

*k*, cluster extent; L., left; R., right.

**Table 2:** Comparison of PPI of VOIs between maternal and grandmaternal caregiving groups during digit n-back task (2-back > 0-back).

Seed	Brain Region	MNI Coordinate			*k*	*t*
		*x*	*y*	*z*		
R. Middle frontal gyrus						
	L. Middle cingulate gyrus	−8	−4	44	772	6.05
	L. Heschl gyrus	−54	−16	8	465	5.15
L. Superior frontal gyrus						
	L. Postcentral gyrus	−22	−38	56	7948	7.09
	L. Lingual gyrus	−10	−54	0	3322	6.74
	R. Middle temporal gyrus	52	−44	8	123	5.15
	L. Superior occipital gyrus	−10	−84	44	547	4.96
	L. Middle temporal gyrus	−50	−68	0	238	4.44

*k*, cluster extent; L., left; R., right.

**Table 3:** Brain activation during negative emotional face task (emotional face > scrambled face) across all participants.

Brain Region	MNI Coordinate			*k*	*t*
	*x*	*y*	*z*		
R. Inferior occipital gyrus	32	−92	10	1696	15.17
L. Middle occipital gyrus	−24	−94	0	1051	14.21
R. Opercular part of the inferior frontal gyrus	42	14	28	658	12.00
L. Precentral gyrus	−42	6	30	181	7.67

*k*, cluster extent; L., left; R., right.

**Table 4:** Comparison of PPI of VOIs between maternal and grandmaternal caregiving groups during negative emotional face task (emotional face > scrambled face).

Seed	Brain Region	MNI Coordinate			*k*	*t*
		*x*	*y*	*z*		
R. Inferior occipital gyrus						
	L. Rolandic operculum	−42	−32	18	551	5.03
	L. Orbital part of the inferior frontal gyrus	−38	20	−12	324	4.84
	R. Superior temporal gyrus	46	−38	14	305	4.84
	L. Superior temporal gyrus	−58	−22	6	249	4.50
L. Precentral gyrus						
	R. Lingual gyrus	22	−54	−8	258	6.02
	R. Putamen	30	−16	2	176	4.41

*k*, cluster extent; L., left; R., right.

## Data Availability

The data that support the findings of this study are available from the corresponding author upon reasonable request.
